# Bibliometric Analysis of γδ T Cells as Immune Regulators in Cancer Prognosis

**DOI:** 10.3389/fimmu.2022.874640

**Published:** 2022-04-14

**Authors:** Bing Liu, Xu He, Yong Wang, Jian-wen Huang, You-bing Zheng, Yong Li, Li-gong Lu

**Affiliations:** Department of Interventional Medicine, Zhuhai People’s Hospital (Zhuhai Hospital affiliated with Jinan University), Zhuhai, China

**Keywords:** bibliometric analysis, γδ T cells, cancer prognosis, immune regulators, IL-17, IL-2

## Abstract

γδ T cells are one of only three immune cell types that express antigen receptors that undergo somatic recombination, and they contribute to immune responses to infection, cellular transformation, and tissue damage. As a “bridge” between the innate and adaptive immune systems, γδ T cells have been noted to be involved in various immune responses during cancer progression. The purpose of our study was to review current published information on γδ T cells and investigate their functions in different types of malignancy using bibliometric and bioinformatic methods. Our results indicated that studies on γδ T cells and cancer progression increased from 2014, and the number had peaked by 2021. We discovered that there is international cooperation in the performance of studies among 26 countries, where China was identified as the most productive with the highest citations. Using keyword co-occurrence analysis, we found that among all the cancer types investigated, gastric and breast cancers were most closely related to γδ T cells. Furthermore, interleukin (IL)-17 and IL-2 were the most common cytokines linked to γδ T cells and our investigation of their potential involvement in the prognosis of gastric and breast cancers, identified their different roles in various malignancies. Thus, we concluded that γδ T cells might influence the progression of different cancers in diverse ways.

## Introduction

T lymphocytes play a critical role in the response and regulation of human immune functions (1). Human T lymphocytes can be divided into αβ (such as CD4 and CD8) and γδ T cells according to the T cell receptor (TCR) structures. In human peripheral blood lymphocytes, αβ T cells are the dominant cells, whereas γδ T cells generally account for only 1% to 5% (2). The functions of these two types of cells and their response mechanisms in the immune response are also different. For example, the recognition of antigens by αβ T but not γδ cells depends on major histocompatibility complex (MHC) molecules (3).

Human γδ T cells have numerous unique biological functions. Based on their distribution and ability to recognize antigens, this population of cells is considered a “bridge” between innate and adaptive immunity (4). According to the structural differences between the γ and δ chains, γδ T cells can be divided into two main subgroups, namely Vδ1 and Vδ2, with distinct functions (5). For example, the Vδ2 cell subset inhibits bacterial infection and tumor progression (6, 7). In human peripheral blood, 50–90% of γδ T cells express Vγ9Vδ2 receptors (8). An increasing number of studies have shown that Vγ9Vδ2 T cells have a very important inhibitory effect on the occurrence and development of tumors by significantly inhibiting the growth of tumor cells (9).

However, a pro-tumor role for interleukin (IL)-17-producing γδ T cells has also been reported in human cancers. Specifically, Vδ1 T cells are the major source of the IL-17 involved in chronic inflammation in colorectal cancer (10). These results indicate that γδ T cells might be a “double-edged sword” in cancer treatment. In this study, we aimed to summarize the current knowledge of γδ T cell research and investigate their functions in different subtypes of malignancy using bibliometrics and bioinformatics methods.

## Methods

### Scopus Search

Scopus (Elsevier, Amsterdam, The Netherlands) was chosen as the main database for our literature search (11, 12). The following search formulas were used in Scopus: TITLE-ABS-KEY (“γδT cell” and “cancer”) AND PUBYEAR>1993 AND PUBYEAR<2022. To avoid citation duplication, the literature search and extraction were completed on a single day, January 10, 2022 and the result yielded 239 studies.

The titles, abstracts, and keywords of the 239 studies were scanned and filtered manually. Full texts were further examined where necessary. To achieve precise and non-duplicated results, we set the inclusion criteria as (1) a clear correlation between γδT cell and cancer; (2) human, mouse, or cell-based studies; and (3) document type as “article”. Finally, 190 studies were included and summarized in a csv file for the subsequent analysis.

### Analysis Using VOSviewer

We uniformed “γδt cell, γδt cells, γδt-cell, γδt-cells, gdT cells, gamma delta T cell, gamma delta T cells, and gamma delta T lymphocyte” to “γδ T cell”. In addition, “natural killer cell, natural killer cells, nkt cell, and nkt cells” were uniformed to “nk cells”. Then, the csv file was uploaded into VOSviewer to conduct co-occurrence analysis of authors, countries, and keywords. The minimum number of occurrences of each keyword was set to five and the total intensity of co-occurrence bonds to other keywords was also derived.

### Overall Survival Estimation of γδ T Cell in Different Malignancies

The Kaplan–Meier plotter (13) is an online tool for assessing the correlation between the expression of 30 k genes (mRNA, miRNA, and protein) and survival in 25 k+ samples from 21 tumor types. We conducted a survival analysis of γδT cells with different subtypes of cancer. Based on the median transcription level of each target gene, patients were allocated to the high and low expression groups, and Kaplan–Meier plots were generated accordingly. The hazard ratio (HR) with the 95% confidence interval and log-rank p-values were also calculated. Statistical significance was set at p < 0.05.

## Results

**Figure 1:**
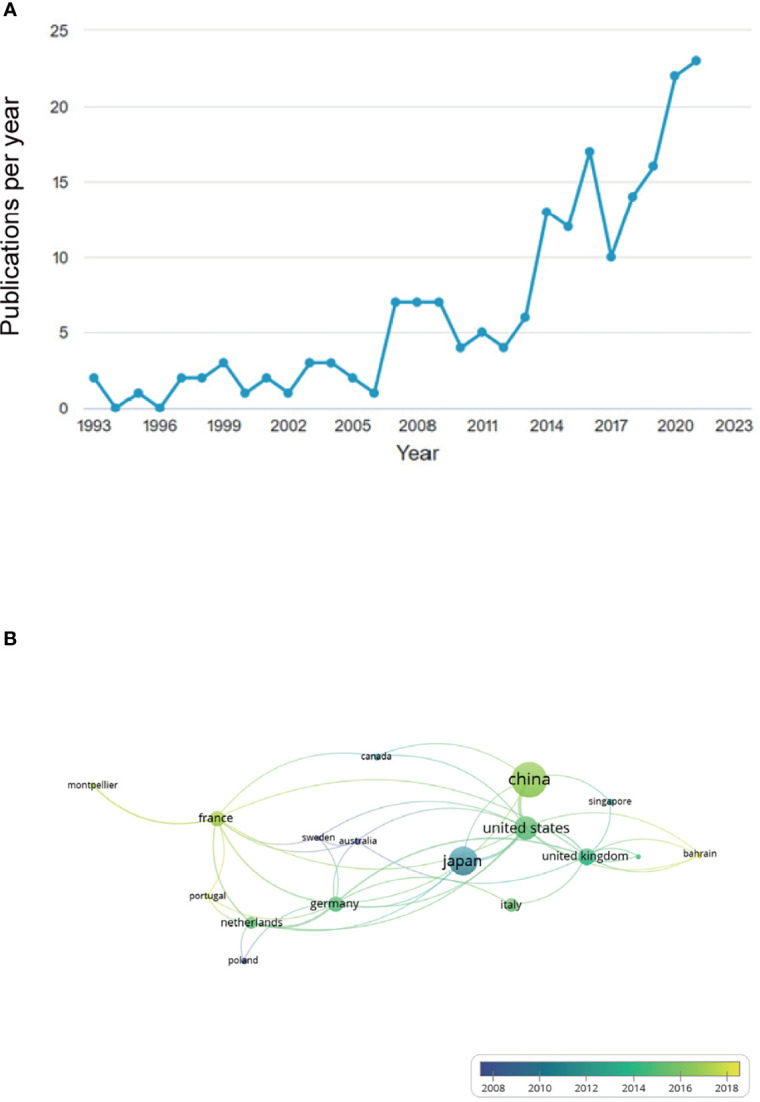
Annual number of publications related to γδ T cell and cancer increased from 2014 to 2021 and international cooperation in related research occurred between different countries. **(A)** Before 2006, there were few studies on γδ T cell and cancer progression, annually, but number of studies increased from 2014 and peaked by 2021. **(B)** Numerous national connections were established in this field of study, with China, Japan, and the US emerging as the most productive countries.

Our literature search identified 190 studies on the relationship between γδT cells and cancer conducted from 1993 to 2021. The results indicated that before 2006, there were few studies on γδ T cells and cancer progression annually, whereas the number increased from 2014 and had peaked by 2021 (**Figure 1A**). There was international cooperation in conducting studies among 26 countries, and China had the most publications as well as the highest citations, followed by Japan and the US (**Figure 1B**). The top-20 cited publications are listed in **Table 1**. The paper “*Enterococcus hirae* and *Barnesiella intestinihominis* facilitate cyclophosphamide-induced therapeutic immunomodulatory Effects” by Daillère et al. (13) had the highest citations at 312 times. The publication “γδT17 cells promote the accumulation and expansion of myeloid-derived suppressor cells in human colorectal cancer” by Wu et al. (10) had the second highest number of citations at 301 times.

**Table 1:** Top 20 cited publications studying relationship between γδ T cell and cancer.

Authors	Title	Year	Source title	Cited by	PubMed ID
Daillère et al.	Enterococcus hirae and Barnesiella intestinihominis Facilitate Cyclophosphamide-Induced Therapeutic Immunomodulatory Effects	2016	Immunity	312	27717798 (14)
Wu et al.	γδT17 cells promote the accumulation and expansion of myeloid-derived suppressor cells in human colorectal cancer	2014	Immunity	301	24816404 (10)
Daley et al.	γδ T Cells Support Pancreatic Oncogenesis by Restraining αβ T Cell Activation	2016	Cell	168	27569912 (15)
Sato et al.	Cytotoxic effects of γδ T cells expanded ex vivo by a third generation bisphosphonate for cancer immunotherapy	2005	International Journal of Cancer	136	15756684 (16)
Schnurr et al.	Apoptotic pancreatic tumor cells are superior to cell lysates in promoting cross-priming of cytotoxic T cells and activate NK and γδT cells	2002	Cancer Research	127	11956095 (17)
Kong et al.	The NKG2D ligand ULBP4 binds to TCRγ9/δ2 and induces cytotoxicity to tumor cells through both TCRγδ and NKG2D	2009	Blood	120	19436053 (18)
Alexander et al.	Isopentenyl pyrophosphate-activated CD56+ γδ T lymphocytes display potent antitumor activity toward human squamous cell carcinoma	2008	Clinical Cancer Research	105	18594005 (19)
Sakamoto et al.	Adoptive immunotherapy for advanced non-small cell lung cancer using zoledronate-expanded γδT cells: A Phase I clinical study	2011	Journal of Immunotherapy	102	21304399 (20)
Laurent et al.	The engagement of CTLA-4 on primary melanoma cell lines induces antibody-dependent cellular cytotoxicity and TNF-α production.	2013	Journal of translational medicine	97	23634660 (21)
Grose et al.	The role of fibroblast growth factor receptor 2b in skin homeostasis and cancer development	2007	EMBO Journal	95	17304214 (22)
Capsomidis et al.	Chimeric Antigen Receptor-Engineered Human Gamma Delta T Cells: Enhanced Cytotoxicity with Retention of Cross Presentation	2018	Molecular Therapy	77	29310916 (23)
Okada et al.	Origin of CD57+ T cells which increase at tumour sites in patients with colorectal cancer	1995	Clinical and Experimental Immunology	77	7554383 (24)
Cui et al.	Combination of radiofrequency ablation and sequential cellular immunotherapy improves progression-free survival for patients with hepatocellular carcinoma	2014	International Journal of Cancer	69	23825037 (25)
Sudam Patil et al.	IL17 producing γδT cells induce angiogenesis and are associated with poor survival in gallbladder cancer patients	2016	International Journal of Cancer	67	27062572 (26)
Legut et al.	The promise of γδT cells and the γδT cell receptor for cancer immunotherapy	2015	Cellular and Molecular Immunology	64	25864915 (27)
Marcu-Malina et al.	Redirecting αβT cells against cancer cells by transfer of a broadly tumor-reactive γδT-cell receptor	2011	Blood	60	21566093 (28)
Ni et al.	Breast cancer-derived exosomes transmit lncRNA SNHG16 to induce CD73+γδ1 Treg cells	2020	Signal Transduction and Targeted Therapy	55	32345959 (29)
Fisher et al.	Neuroblastoma killing properties of Vδ2 and Vδ2-negative γδT cells following expansion by artificial antigen-presenting cells	2014	Clinical Cancer Research	52	24893631 (30)
Holderness et al.	Select plant tannins induce IL-2Rα up-regulation and augment cell division in γδ T cells	2007	Journal of Immunology	45	17982035 (31)
Hu et al.	Tumor-infiltrating CD39+ γδTregs are novel immunosuppressive T cells in human colorectal cancer	2017	OncoImmunology	43	28344891 (32)

**Figure 2:**
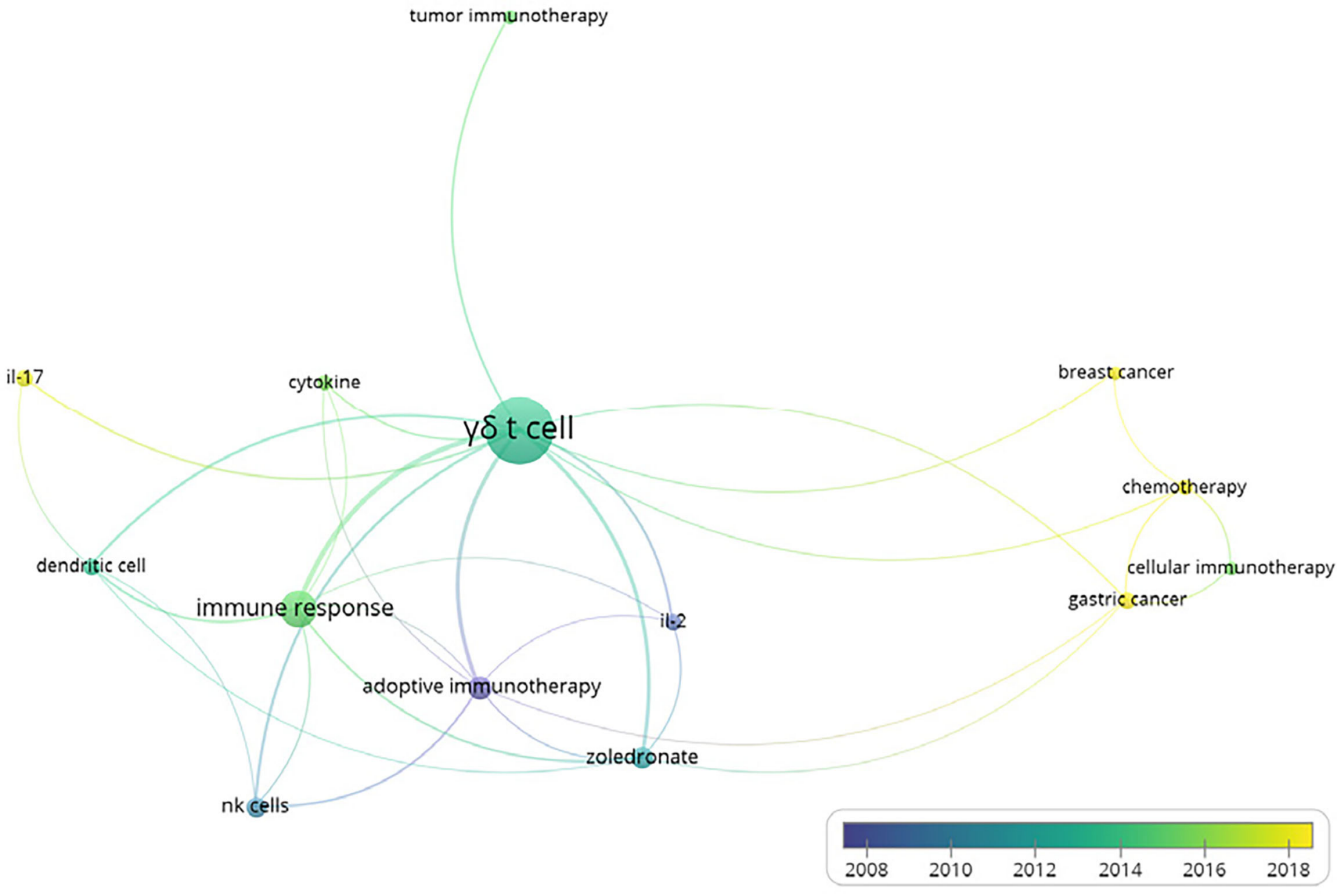
Overlay visualization map of author keywords co-occurrence analysis. Map shows that γδ T cells mediated immune responses. Of all investigated cancers, gastric and breast cancers were most closely linked to γδ T cells. Interleukin (IL)-17 and IL-2 were identified as the most common cytokines linked to γδ T cells.

Furthermore, we also conducted the co-occurrences analysis of keywords with the software VOSviewer, in order to figure out the relationship between γδT cells and other important scientific issues. The minimum number of occurrences of a keyword was set to 5 and 14 were finally identified among all 564 keywords. The hotspots in γδT cell functions are presented in the overlay visualization map scaled by occurrences (**Figure 2**). Accordingly, γδT cells were shown to be responsible for the immune response and communicated with dendritic cells and NK cells. We also found that among all the types of cancer, gastric and breast cancer were most closely linked to γδ T cells. Furthermore, IL-17 and IL-2 are the most common cytokines linked to γδ T cells.

IL-17 production by certain γδ T cell subsets has been reported to recruit immunosuppressive cells such as myeloid-derived suppressor cells (MDSCs) or small peritoneal macrophages, which can promote angiogenesis, tumor cell growth, and inducible regulatory T (Treg) cell differentiation (7). IL-2 could augment the γδT-17 response in favor of short-lived effectors with limited plasticity, particularly in the presence of IL-1β and IL-23 (33). Taken together, these results led us to conclude that γδ T cells could have a role in cancer development *via* IL-2 or IL-17. Consequently, we investigated the effects of IL-17 and IL-2 on gastric and breast cancer, and identified their distinct roles in different malignancies.

**Figure 3:**
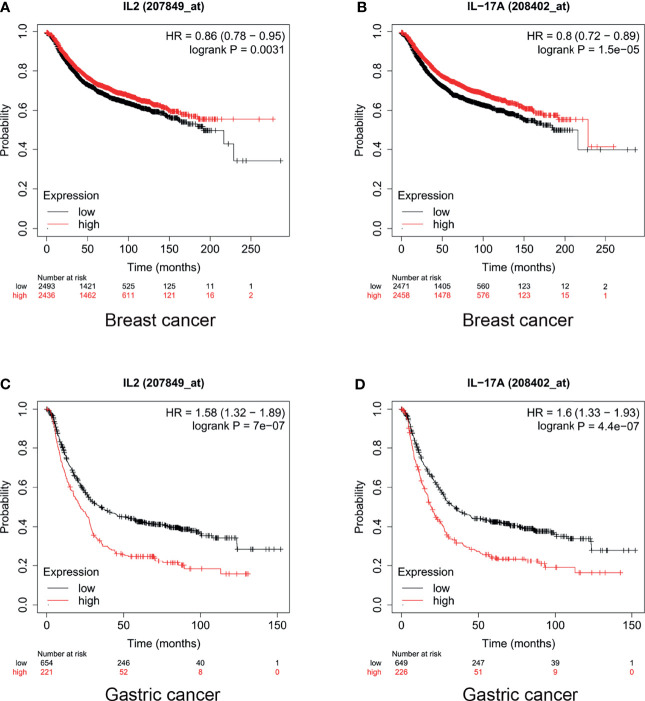
Levels of interleukin (IL)-2 and IL-17 played different roles in breast and gastric cancer prognosis. High IL-17 and IL-2 expression indicated **(A, B)** promising prognosis in breast cancer and **(C, D)** poor prognosis in gastric cancer.

In breast cancer, high expression levels of IL-17 and IL-2 indicated a promising prognosis. The median survival of patients with breast cancer with low and high expression levels of IL-2 was 43 months and 56 months, respectively (HR = 0.86, p = 0.0031, **Figure 3A**). Furthermore, the median survival of breast cancer patients with low and high expression levels of IL-17 was 216.66 and 228.85 months, respectively (HR = 0.8, p < 0.001, **Figure 3B**). However, IL-2 and IL-17 played a dinstinct role in gastric cancer prognosis. Our results also showed that the median survival of patients with gastric cancer who had low and high expression levels of IL-2 was 35.4 and 22 months, respectively (HR = 1.58, p < 0.001, **Figure 3C**). In addition, for patients with gastric cancer with low and high expression levels of IL-17, the median survival was 34.7 and 19.5 months (HR = 1.6, p < 0.001, **Figure 3D**).

**Figure 4:**
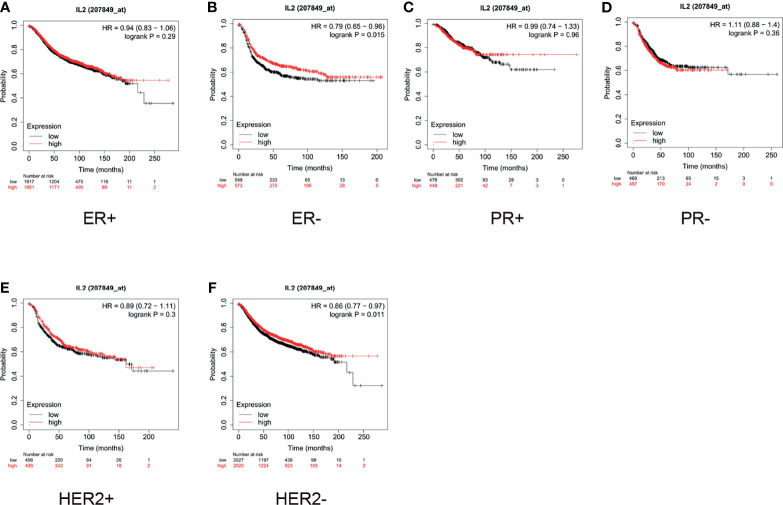
In patients with breast cancer, high interleukin (IL)-2 expression only indicated better prognosis in estrogen receptor-negative (ER-) and human epidermal growth factor receptor 2 negative (HER2-) subgroups. **(A)** High expression of IL-2 **(A)** did not correlate with better prognosis in ER+ patients (hazard ratio [HR] = 0.94, p = 0.29) and **(B)** indicated better prognosis in ER- patients (HR = 0.79, p = 0.015). **(C, D)**. IL-2 expression did not significantly influence survival in progesterone receptor-positive (PR+; HR = 0.99, p = 0.96) or PR- (HR = 1.11, p = 0.36) subgroups. High expression of IL-2 **(E)** did not correlate with better prognosis in HER2+ patients (HR = 0.89, p = 0.3) and **(F)** indicated better prognosis in HER2- patients (HR = 0.86, p = 0.011).

Furthermore, we conducted a subgroup analysis of these two types of cancers. For breast cancer, we examined the status of the estrogen receptor (ER), progesterone receptor (PR), and human epidermal growth factor receptor 2 (HER2). As shown in **Figure 4**, the median survival of ER-negative (ER-) patients with low expression of IL-2 was 18 months, whereas that of patients with high expression was 25 months (HR = 0.79, p = 0.015, **Figure 4B**). For HER2- patients, the median survival of patients with low expression of IL-2 was 50 months, whereas that of patients with high expression was 61.92 months (HR = 0.86, p = 0.011, **Figure 4F**). The expression of IL-2 did not influence survival in ER-positive (ER+), HER2+, PR+, or PR- subgroups (**Figures 4A, C–E**).

**Figure 5:**
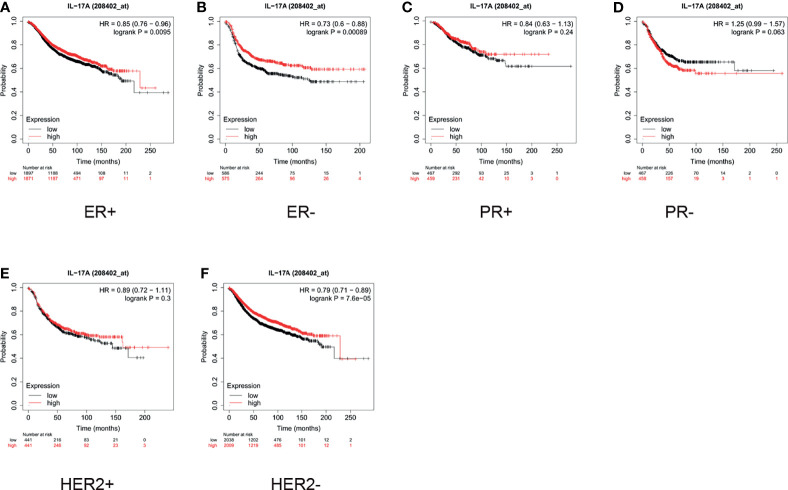
In breast cancer patients, high expression of interleukin (IL)-17 only indicated better prognosis in estrogen receptor-positive (ER+), ER-negative (ER-), and human epidermal growth factor receptor 2 negative (HER2-) subgroups. High expression of IL-17 indicated better prognosis in **(A)** ER+ (hazard ratio [HR] = 0.85, p < 0.001) and **(B)** ER- (HR = 0.73, p < 0.001) patients. **(C, D)** IL-17 expression did not significantly influence survival in progesterone-positive (PR+; HR = 0.84, p = 0.24) or PR- (HR = 1.25, p = 0.063) subgroups. **(E)** High expression of IL-17 did not correlate with better prognosis in HER2+ patients (HR = 0.89, p = 0.3). **(F)** High expression of IL-2 indicated better prognosis in HER2- patients (HR = 0.79, p < 0.001).

As shown in **Figure 5**, for ER+ patients, high expression of IL-17 was significantly correlated with a longer survival time (HR = 0.85, p = 0.0095, **Figure 5A**). For ER- patients, the median survival of those with low and high expression of IL-17 was 18 and 28.75 months, respectively (HR = 0.73, p = 0.0009, **Figure 5B**). The expression of IL-17 did not significantly influence the overall survival, regardless of whether the patients were PR+ or PR- (**Figures 5C, D**). As demonstrated in **Figure 5E**, high expression of IL-17 did not correlate with better prognosis in HER2+ patients (HR = 0.89, p = 0.3). For HER2- patients, high expression of IL-17 indicated a better prognosis than low expression did (HR = 0.79, p < 0.001, **Figure 5F**).

**Figure 6:**
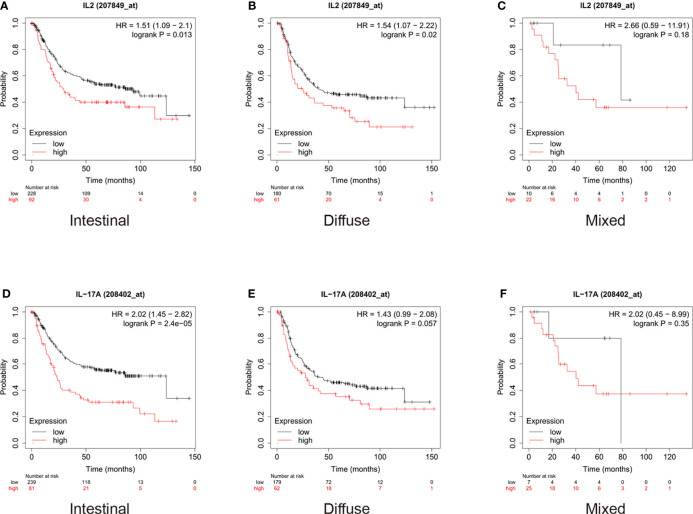
In gastric cancer patients, high expression of interleukin (IL)-2 indicated worse prognosis in patients with both intestinal and diffuse conditions and high expression of IL-17 indicated worse prognosis only in patients with intestinal condition. **(A, B)** High expression of IL-2 indicated worse prognosis in patients with both intestinal (hazard ratio [HR] = 1.51, p = 0.013) and diffuse (HR = 1.54, p = 0.3) conditions. **(C)** High expression of IL-2 did not significantly influence survival in mixed patients (HR = 2.66, p = 0.18). **(D)** High expression of IL-17 indicated worse prognosis in patients with intestinal conditions (HR = 2.02, p < 0.001). **(E, F)** High expression of IL-17 did not significantly influence survival in patients with diffuse (HR = 1.43, p = 0.057) and mixed (HR = 2.02, p = 0.35) conditions.

For gastric cancer, we used the Lauren classification to divide the cohort into three subgroups: intestinal, diffuse, and mixed. As shown in **Figure 6**, the analysis of the expression of IL-2 in different subtypes of gastric cancer showed that low expression of IL-2 indicated a better prognosis in both intestinal and diffuse patients than high expression levels did (**Figures 6A, B**). However, the difference was not statistically significant in patients with mixed conditions (**Figure 6C**). Moreover, the median survival of intestinal patients with low expression of IL-17 was 123.8 months, whereas that of patients with high expression was only 23.4 months (HR = 2.02, p < 0.001, **Figure 6D**), and the difference did not have statistical significance in diffuse and mixed subgroups (**Figures 6E, F**).

## Discussion

Our results showed that the number of studies on γδ T cells has increased since 2014, suggesting that most were likely novel. The examination of the top-20 cited publications revealed that numerous studies concentrated on the role of γδ T cells in cancer treatment. In addition, the co-occurrence analysis revealed that γδ T cells were more closely related to breast and gastric cancers than they were to the other investigated malignancies. IL-2 and IL-17 are the two most important cytokines related to γδ T cells; therefore, we investigated their influence on breast and gastric cancers.

The two cytokines, IL-2 and IL-17, which we focused on in this study, play different roles in breast and gastric cancers, where they promote the development of gastric cancer but inhibit the progression of breast cancer. The results of the subgroup analysis further clarified this finding. For intestinal gastric cancer, low expression of IL-2 and IL-17 indicated a promising prognosis, whereas for diffuse and mixed gastric cancers, the expression of these cytokines did not significantly affect survival. The cases of intestinal gastric cancer were deemed to be early stage and, therefore, we speculated that γδ T cells might affect the prognosis of gastric cancer at the disease onset.

In breast cancer, ER, PR, and HER2 status have been verified as important prognostic factors; therefore, based on these different statuses, we also investigated how the expression of IL-2 and IL-17 influenced patient survival. Our results showed that regardless of ER status, high expression of IL-17 indicated a longer survival time, but only a better prognosis in HER2- patients than low expression did. In addition, high expression of IL-2 indicated a better prognosis in patients with ER- or HER2- breast cancer than low expression did. However, regardless of whether PR was positive or negative, the expression of IL-2 and IL-17 did not influence the survival of patients with breast cancer in this subgroup analysis. Based on these results, we hypothesized that HER2 might play an important role in breast cancer treatment *via* γδ T cells.

IL-2 and IL-17 have opposite roles in the development of breast and gastric cancers and, therefore, we postulated that γδ T cells have distinct functions in different malignancies. Furthermore, we also discovered that even in the same malignancy, γδ T cells might have distinct functions in different forms, implying some underlying mechanism may exist between γδ T cells and cell receptors. The abundant cytokine secretion and non-MHC-restricted antigen recognition capacity of γδ T cells has encouraged the investigation of their application in cancer adoptive immunotherapy (34). Currently, evidence has accumulated from studies in numerous cancers, and the results demonstrate that γδ T cells could be well tolerated in the treatment of cancer (3, 34–36). Studies have also revealed that γδ T cells can exert anticancer activity through various mechanisms, such as eliminating tumor cells *via* the perforin-granzyme pathway (37), binding to TNF-related apoptosis-inducing ligand (TRAIL) and Fas ligand (FasL) (38), *via* antibody-dependent cellular cytotoxicity (ADCC) (39), or by secreting interferon (IFN)-γ and tumor necrosis factor (TNF)-α (40, 41). In addition to these direct antitumor effects, specific γδ T cell subsets also exert an indirect antitumor effect, which is complemented by interactions with other immune cells such as B cells, DCs, αβ T cells, and NK cells (42). However, recent studies claim that γδ T cells could stimulate cancer development (43–45) by impairing the antitumor ability of immunocytes or enhancing the function of immunosuppressive cells (10, 46, 47). For instance, γδ T17 cells are a major source of IL-17 in the cancer microenvironment (48), and IL-17 contributes to cancer development by supporting angiogenesis in several malignancies, such as gallbladder cancer, gastric cancer and non-small-cell lung cancer (26, 49, 50).

The limitation of the current study was that we only chose Scopus as our database and did not use databases such as Pubmed or Google Scholar. Besides, as we used the Kaplan Meier plotter as our database to achieve survival data, this database collected information from many independent datasets, which might cause bias in our analysis.

**Figure 7:**
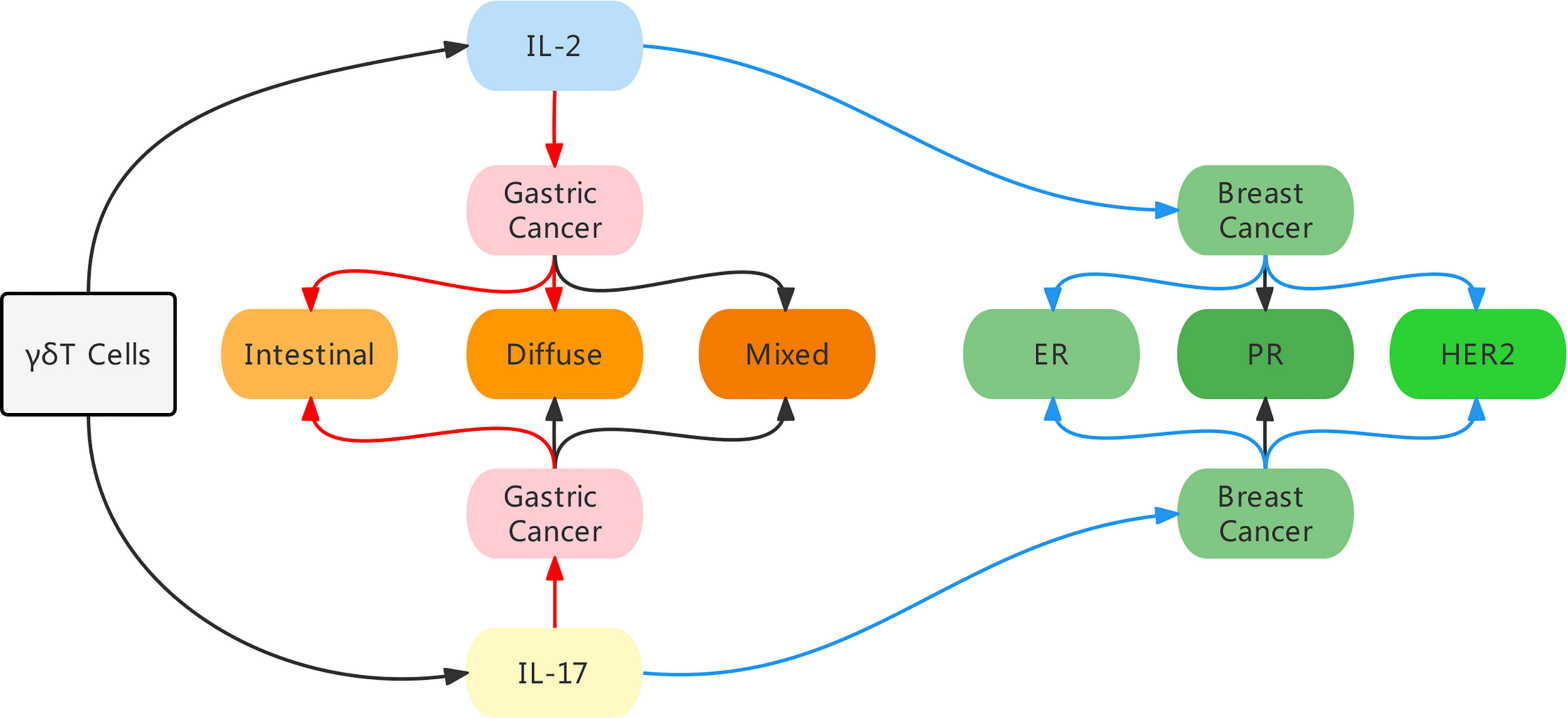
An illustration of the main findings in our study. By bibliometric analysis, we identified IL-17 and IL-2 as the most common cytokines linked to γδ T cells, and these two cytokines influenced the prognosis in different manners in gastric cancer and breast cancer. In breast cancer, high expression of IL-2 and IL-17 indicated a better prognosis, especially in ER negative and HER2 negative patients. While in gastric cancer patients, high level of IL-2 indicated a worse prognosis in intestinal and diffuse conditions, and high level of IL-17 was merely predictive of a poor prognosis in intestinal condition. Red arrow indicates a poor prognosis, blue arrow indicates a good prognosis.

In conclusion, using bibliometric analysis, we identified IL-17 and IL-2 as the most common cytokines linked to γδ T cells. Furthermore, based on our investigation of the role of IL-17 and IL-2 in the prognosis of gastric and breast cancer, we discovered that they play different roles in various malignancies. Moreover, in the same malignancy, the expression levels of certain genes or different variations could impact the function of γδ T cells (**Figure 7**). Finally, we concluded that γδ T cells might influence the progression of different cancers in diverse ways.

## Data Availability Statement

The datasets presented in this study can be found in online repositories. The names of the repository/repositories and accession number(s) can be found in the article/supplementary material.

## Author Contributions

BL conducted the data search and paper writing, XH and YW contributed to the bibliometric analysis, J-wH and Y-bZ revised the figures and tables, YL and L-gL designed the study and revised the paper. All authors contributed to the article and approved the submitted version.

## Funding

This work is partially supported by National Key Research and Development Program of China (No. 2017YFA0205200) and the National Natural Science Foundation of China (No. 81571785, 81771957, 81801811).

## Conflict of Interest

The authors declare that the research was conducted in the absence of any commercial or financial relationships that could be construed as a potential conflict of interest.

## Publisher’s Note

All claims expressed in this article are solely those of the authors and do not necessarily represent those of their affiliated organizations, or those of the publisher, the editors and the reviewers. Any product that may be evaluated in this article, or claim that may be made by its manufacturer, is not guaranteed or endorsed by the publisher.
